# Ether and Chloroform

**Published:** 1872-11

**Authors:** 


### Ether and Chloroform.

The constantly recurring accounts of deaths from chloroform seem to have no terror for its advocates, who doubtless honestly believe these accidents to be due to carelessness in administration, or to impurities in the agent itself. Two cases, indeed, have recently been reported, in which the inhalation of sulpheric ether was accused of fatal results. But even if these were correctly construed, which we doubt, can they weigh at all against the hundreds and hundreds in which the lethal effects of chloroform were beyond question? And has this latter any real advantages to offset its fearful risks? Its advocates say that it is easier of administration, more agreeable to the patient, and less apt to induce nausea than ether; and in certain operations its non-inflammability is a strong point in its favor. And yet our own experience leads us to doubt whether the safer agent is not in most cases quite as available. Dr. Jeffries, of Boston, we are told, amazed the ophthalmic surgeons in London, at the recent Congress, by showing how easily and how perfectly ether answered the purpose of producing anæsthesia for operations on the eye. We should be glad to receive communications on this most important subject, and on that of anæsthetics generally—*Med. Times.*

